# Focus on the Potential Role of Lung Ultrasound in COVID-19 Pandemic: What More to Do?

**DOI:** 10.3390/ijerph17228398

**Published:** 2020-11-13

**Authors:** Beatrice Ragnoli, Mario Malerba

**Affiliations:** 1 Respiratory Unit, S. Andrea Hospital, 13100 Vercelli, Italy; beatrice.ragnoli@aslvc.piemonte.it; 2 Department of Traslational Medicine, Università del Piemonte Orientale, 28100 Novara, Italy

COVID-19, a novel severe acute respiratory syndrome (SARS) emerging in China’s Hubei province in late 2019, due to a new coronavirus (SARS-CoV-2), is causing a global pandemic involving many areas of the world, which so far counts more than 43 million cases and more than 1,155,000 deaths worldwide [1,2]. In Italy, at the time of writing, we are facing a second wave of infections with an important pressure on hospital structures. The virus has a specific tropism for the lower respiratory tract in the early disease stage. About 20% of affected patients are at risk of developing the severe form of the disease with an acute respiratory distress syndrome (ARDS) and with high morbidity/mortality [3]. The histopathologic aspect of initial COVID-19 pneumonia is characterized by alveolar edema and damage while inflammatory component is usually patchy and mild. Reparative processes with pneumocyte hyperplasia and/or interstitial thickening may be present while the late phases show gravitational consolidations, edema, alveolar congestion, hemorrhagic necrosis and fibrosis [4]. Computed tomography (CT) is considered the routine imaging technique for diagnosis and monitoring of COVID-19 pneumonia [5]. Data show a high specificity and sensitivity for diagnosis of COVID-19, even though CT chest is not inexpensive and universally available; moreover, it often requires an infected or unstable patient to be moved to the scanner or radiology unit with potential exposure of several people including staff members, needing proper sanitation of the CT room after use and personal protective equipment; lastly, it is underutilized in children and pregnant women because of concerns over radiation exposure. In this context, considering the increasing frequency of confirmed COVID-19 cases and the need of new, highly sensitive and faster diagnostic tools, the role of lung ultrasound (LUS) has become relevant. LUS or Point-Of-Care Ultrasound (POCUS) is already known and used in different settings including triage, diagnosis, and follow-up of different lung lesions and routinely managed by critical care specialists, emergency physicians, and, recently, internists as an alternative to chest X-ray and CT scan [6] has reached popularity, demonstrating a better diagnostic yield than a chest X-ray in the early diagnosis of H1N1 2009 pandemic viral pneumonia [7]. LUS is a non-invasive bedside technique that, in patients with COVID-19, pneumonia reveals a typical pattern of diffuse interstitial lung syndrome, characterized by multiple or confluent bilateral B-lines with spared areas, thickening of the pleural line with pleural line irregularity and, less frequently, peripheral (subpleural) nodules or consolidations, appearance of A lines during recovery phase while pleural effusion is uncommon [8,9,10]. In these patients, different degrees of interstitial syndrome and alveolar consolidation are directly correlated with the severity of the lung injury [11]. Although currently it is not possible to identify pathognomonic findings of COVID-19, it was recently reported the “light beam” as a broad, band-shaped, vertical artefact that moves rapidly with sliding, corresponding to the early “ground glass” [12]. Moreover, LUS can detect the pulmonary changes associated with the progression of the COVID-19 infection [13]. In early stages, the most frequent ultrasonographic finding are focal B-lines, while during progression of the disease B-lines become multifocal and confluent (interstitial lung syndrome) with development of consolidations, then, during convalescence, B-lines and consolidations gradually reduces and disappear replaced by A-lines [14]. In this setting, LUS findings appear to correlate well with findings on chest CT [15] and it was observed that sensitivity, specificity and diagnostic accuracy of lung ultrasound increase with the severity of COVID-19 pneumonia similarly to chest CT scan [16]. Compared to CT, LUS has several advantages, such as lack of exposure to radiation, bedside repeatability during follow-up, low cost and easier application in low-resource settings. Consequently, LUS may decrease utilization of conventional diagnostic imaging resources (CT scan and chest X-ray). LUS may help in early diagnosis, therapeutic decisions and follow-up monitoring of COVID-19 pneumonia, particularly in the critical care setting and in pregnant women, children and patients in areas with high rates of community transmission [17]. The ease of use of bedside LUS moreover allows integration between lung state information (sonographic pattern) with medical history, risk of exposure, clinical features and blood exams giving the clinicians a better characterization of the disease thus helping in decision-making processes [18]. A multiparametric approach, in fact, may increase the diagnostic accuracy of LUS for COVID-19 pneumonia, especially in mild–moderate disease. Data from studies in different clinical settings may support the role of LUS guiding therapeutic decisions and procedures in COVID-19 patients; in intensive care-units, it is possible to track the evolution of interstitial pneumonia and ARDS, to monitor therapeutic response and to identify early possible complications and to guide invasive procedures (ventilator support) [19]; in emergency departments, for suspected cases to have an immediate evaluation of the lung and to detect early pulmonary findings even in patients without respiratory symptoms/fever especially if pulse oxygen levels are lower than normal values [20]; in pulmonology and general internal medicine units, it allows to monitor the effect of therapeutic drugs as a bedside and real-time technique, and may help to decrease the use of conventional diagnostic imaging resources, reducing exposure of health care workers; in pediatric units, it represents a reliable alternative to CT scan and X-ray; finally, LUS can be used by general practitioners as first step for patients clinically suspected reducing unnecessary emergency department visits and to provide additional data-point to the traditional chest auscultation allowing advanced triage for COVID-19 directly at home [21]. LUS with a portable device has been successfully used as a tool for identifying COVID-19 in nursing home residents, suggesting a potential role for screening patients at high risk in a setting with limited resources.

In conclusion, LUS may be a first-line diagnostic imaging alternative to chest CT scan and chest X-ray during every step of COVID-19 infection, even before clinical manifestations, particularly in children and pregnant women, critically ill patients that cannot be moved and patients in areas with high rates of community transmission and low resources. The combination of sonographic patterns at LUS with clinical and laboratory findings, followed by chest CT for confirmation in selected cases, may help in early diagnosis, therapeutic decisions and follow-up monitoring of COVID-19 pneumonia.

In light if these premises, LUS in clinical practice during COVID pandemic has become an ideal imaging modality to consent an early triaging of patients into mild or moderate and severe pneumonia, even in different settings before hospital admission, where different professional figures with an adequate training can represent a first important step for clinically suspected cases or, on the contrary, to avoid unnecessary hospitalization, especially in case of overcrowding and overloading of health care system, LUS is highly specific for COVID-19 and sensitive in ruling out, allowing repeated examinations that are useful to monitor progression or resolution of the disease even at home, available at the bedside of the patient without bringing additional staff and equipment into contaminated areas or moving infectious patients to clean areas, and it is cheap. In our unit, during the pandemic in March, we used it to identify at admittance the degree of interstitial syndrome severity to better monitor patients progress and as a tool for an early intervention in case of expected worsening. In selected cases, we used it in addition to other tools and sometimes substituting chest radiography when a high risk transmission for staff members involved in the procedure was present. The progress of the patient was pointed-out by a simplified score recorded daily at bedside. The physician attending the visit was equipped by a tool protected with plastic, easily cleaned, easily moved and able to store images. In this way, we were also able to rapidly assess possible complications in patients with a clinical deterioration. An aspect we need more to highlight is to have data regarding the evolution of patients that overcame the infection, to observe eventual chronic findings and possibly follow it during the time. To date, in our outpatient controls, we are following the evolution of ex-COVID-19 pneumonia cases with an integrated panel of clinical, radiological and lung function tests, including LUS to collect different data, although more research in this area is needed. Our experience would suggest that lung ultrasound has much more to offer in this perspective, even though important issues like the relatively new technique and an inter-observer variability due to inexperienced operators need to be passed providing focused training to reach solid skills; from this perspective, COVID-19 represents an unmissable challenge.

## References

[1] Zhu N., Zhang D., Wang W., Li X., Yang B., Song J., Zhao X., Huang B., Shi W., Lu R. (2020). A Novel Coronavirus from Patients with Pneumonia in China, 2019. N. Engl. J. Med..

[2] European Centre for Disease Prevention and Control. https://www.ecdc.europa.eu/en/geographical-distribution-2019-ncov-cases.

[3] Huang C., Wang Y., Li X., Ren L., Zhao J., Hu Y., Zhang L., Fan G., Xu J., Gu X. (2020). Clinical features of patients infected with 2019 novel coronavirus in Wuhan, China. Lancet.

[4] Tian S., Hu W., Niu L., Liu H., Xu H., Xiao S.-Y. (2020). Pulmonary Pathology of Early-Phase 2019 Novel Coronavirus (COVID-19) Pneumonia in Two Patients with Lung Cancer. J. Thorac. Oncol..

[5] Bai H.X., Hsieh B., Xiong Z., Halsey K., Choi J.W., Tran T.M.L., Pan I., Shi L.-B., Wang D.-C., Mei J. (2020). Performance of Radiologists in Differentiating COVID-19 from Non-COVID-19 Viral Pneumonia at Chest CT. Radiology.

[6] Laursen C.B., Sloth E., Lassen A., Christensen R.D., Lambrechtsen J., Madsen P.H., Henriksen D.P., Davidsen J.R., Rasmussen F. (2014). Point-of-care ultrasonography in patients admitted with respiratory symptoms: A single-blind, randomised controlled trial. Lancet Respir. Med..

[7] Testa A., Soldati G., Copetti R., Giannuzzi R., Portale G., Gentiloni-Silveri N. (2012). Early recognition of the 2009 pandemic influenza A (H1N1) pneumonia by chest ultrasound. Crit. Care.

[8] Erika P., DaCrema A., Bastoni D., Tinelli V., DeMichele E., Ramos P.M., Marcianò T., Silva M., Vercelli A., Magnacavallo A. (2020). Can Lung US Help Critical Care Clinicians in the Early Diagnosis of Novel Coronavirus (COVID-19) Pneumonia?. Radiology.

[9] Buonsenso D., Pata D., Chiaretti A. (2020). COVID-19 outbreak: Less stethoscope, more ultrasound. Lancet Respir. Med..

[10] Mohamed M.F.H., Al-Shokri S., Yousaf Z., Danjuma M., Parambil J.V., Mohamed S., Mubasher M., Dauleh M.M., Hasanain B., AlKahlout M.A. (2020). Frequency of Abnormalities Detected by Point-of-Care Lung Ultrasound in Symptomatic COVID-19 Patients: Systematic Review and Meta-Analysis. Am. J. Trop. Med. Hyg..

[11] Xing C., Li Q., Du H., Kang W., Lian J., Yuan L. (2020). Lung ultrasound findings in patients with COVID-19 pneumonia. Crit. Care.

[12] Volpicelli G., Gargani L. (2020). Sonographic signs and patterns of COVID-19 pneumonia. Ultrasound J..

[13] Soldati G., Smargiassi A., Inchingolo R., Buonsenso D., Perrone T., Briganti D.F., Perlini S., Torri E., Mariani A., Mossolani E.E. (2020). Is There a Role for Lung Ultrasound During the COVID -19 Pandemic?. J. Ultrasound Med..

[14] Fiala M. (2020). Ultrasound in COVID-19: A timeline of ultrasound findings in relation to CT. Clin. Radiol..

[15] Ottaviani S., Franc M., Ebstein E., DeMaria L., Lheure C., Debray M., Khalil A., Crestani B., Borie R., Dieudé P. (2020). Lung ultrasonography in patients with COVID-19: Comparison with CT. Clin. Radiol..

[16] Lu W., Zhang S., Chen B., Chen J., Xian J., Lin Y., Shan H., Su Z.-Z. (2020). A Clinical Study of Noninvasive Assessment of Lung Lesions in Patients with Coronavirus Disease-19 (COVID-19) by Bedside Ultrasound. Ultraschall Med. Eur. J. Ultrasound.

[17] Allinovi M., Parise A., Giacalone M., Amerio A., Delsante M., Odone A., Franci A., Gigliotti F., Amadasi S., Delmonte D. (2020). Lung Ultrasound May Support Diagnosis and Monitoring of COVID-19 Pneumonia. Ultrasound Med. Biol..

[18] Soldati G., Smargiassi A., Inchingolo R., Buonsenso D., Perrone T., Briganti D.F., Perlini S., Torri E., Mariani A., Mossolani E.E. (2020). On Lung Ultrasound Patterns Specificity in the Management of COVID-19 Patients. J. Ultrasound Med..

[19] Mojoli F., Mongodi S., Orlando A., Arisi E., Pozzi M., Civardi L., Tavazzi G., Baldanti F., Bruno R., Iotti G.A. (2020). Our recommendations for acute management of COVID-19. Crit. Care.

[20] Volpicelli G., Lamorte A., Villén T. (2020). What’s new in lung ultrasound during the COVID-19 pandemic. Intensiv. Care Med..

[21] Shokoohi H., Duggan N.M., Sánchez G.G.-D.-C., Torres-Arrese M., Tung-Chen Y. (2020). Lung ultrasound monitoring in patients with COVID-19 on home isolation. Am. J. Emerg. Med..

